# Advances in two-photon imaging for monitoring neural activity in behaving mice

**DOI:** 10.3389/fnins.2025.1597151

**Published:** 2025-08-11

**Authors:** Ruifeng Li, Nan Li, Dongju Zhu, Kai Shi, Shasha Shen, Yi Zhang

**Affiliations:** ^1^Institute of Neuroscience, College of Medicine, Panzhihua University, Panzhihua, China; ^2^The Functional Science Laboratory, College of Medicine, Panzhihua University, Panzhihua, China

**Keywords:** neuroscience, behaving mice, two-photon imaging technology, indicator, advances

## Abstract

An essential goal in neuroscience is to establish a link between animal behavior and neural activity. Monitoring neural activity during behavior is enhanced by establishing simple and consistent behaviors associated with perceptual cues. In recent years, significant strides have been made in studying the neural activity of behaving mice, thanks to the concurrent advancements in imaging systems and the development of multiple indicators. This review summarizes the current applications and methodological advancements of two-photon imaging technology in the context of behavioral mouse studies and explores the broader potential for applying this technology in future research with behaving mice.

## 1 Introduction

Neuroscience aims to decipher the neural activities underlying behavior from the macroscopic scale down to the level of dendritic spines (Komiyama et al., 2010). This quest began in the late 16th century when Janssen of the Netherlands crafted the prototype of the microscope, enabling the observation of the microscopic world. It continued into the early 20th century, as Cajal initiated modern neuroscience research with the aid of a new generation of optical microscopes. These microscopes, characterized by uniform refractive indices and developed in partnership with Abbe and Otto, were pivotal in advancing our understanding of the micro-world and in the significant development of modern neuroscience research, thanks to breakthroughs in observational techniques. In 1952, Oatley’s invention of scanning electron microscopy marked a significant advancement, facilitating subcellular biological research. However, this method was constrained by its requirement for a vacuum environment, limiting its use to structural studies of ex vivo specimens. Observing neurons in living cells in awake animals presents a considerable challenge in cognitive research. In 1990, Denk revolutionized the field with the invention of the two-photon imaging microscope (Denk et al., 1990), integrating Mayer’s two-photon excitation principle, proposed six decades earlier, with contemporary laser confocal microscopy. This technology provided an unprecedented convenience for examining cellular morphology and function at the resolution level of a single cell *in vivo*. In 2003, the Konnerth research group introduced the Multicell Bolus Loading technique (Stosiek et al., 2003), which allowed for the study of the physiology and diseases of living animals at the neural network level. Concurrently, the ongoing development of genetically encoded calcium indicators (GECIs) has significantly advanced the investigation of brain networks involving different neuronal types at the mesoscale within the intact brain. Moreover, it has enabled the long-term observation of neurodynamic changes in neurons (O'Connor et al., 2009).

In the last century, most neuroscience research on behavioral states relied on blind electrode recordings (Evarts, 1968). Although these methods offered high temporal resolution and spatial resolution reaching several hundred micrometers, it cannot provide spatial visualization capabilities. Early studies at the single-cell level concentrated on ex vivo electrophysiological recordings from brain slices (Stuart and Sakmann, 1994). To achieve stable imaging, early two-photon imaging experiments were mostly performed on anesthetized animals. However, anesthesia greatly reduced overall neural activity (Berg-Johnsen and Langmoen, 1992), and induced synchronization and oscillation (Cheung et al., 2001; Volgushev et al., 2006), which are significantly different from the neural activity in the awake state. To avoid the side effects of anesthesia, two-photon functional imaging studies of awake or behaving mice have increased in the last decade. This upsurge is attributed to the concurrent advancements in two-photon microscopy imaging technology, multiple fluorescent indicators for neuronal activity (e.g., chemically synthesized calcium indicators, genetically encoded calcium indicators), and various behavioral paradigms over the past decade, which together provide a feasible way for exploring neural activity under animal behavioral states (Dombeck et al., 2007).

In the past era, a series of significant studies illuminated our understanding of neural activity in the cerebral cortex under various behavioral states. This article is dedicated to summarizing the technological advancements made thus far. It also aims to explore the broader application prospects of two-photon imaging technology for future research on behaving mice. Furthermore, it delves into the feasibility of employing two-photon microscopy to investigate neural activities within the context of specific behavioral paradigms (Lin and Schnitzer, 2016; Wang H. et al., 2018; Sabatini and Tian, 2020).

## 2 Two-photon imaging on neural activity characteristics in behavioral mice

At both cellular (Kerr and Denk, 2008) and subcellular resolution (Chen et al., 2011) scales, two-photon microscopy is extensively utilized for examining the dynamic changes of cortical neurons in the brain. Hundreds of neurons in a two-dimensional field of view (300 × 300 μm) can be scanned by standard two-photon microscopes at 40 Hz. There are several advantages below (Svoboda and Yasuda, 2006): First, the near-infrared excitation light of the two-photon microscope penetrates tissues more effectively and is associated with less self-fluorescence absorption. Second, the nonlinear excitation mode, coupled with a femtosecond-level excitation time window, results in a more focused excitation area and reduced laser energy. These features help minimize tissue damage, enabling long-term imaging of the sample plane. Consequently, two-photon imaging technology has emerged as one of the most widely used methods for studying the functional microcircuits of neurons at mesoscopic level *in vivo*.

Compared with multi-channel single-unit recording (Wang et al., 2024) and optic fiber based recording (Yao et al., 2018) for studying behavioral animals, two-photon imaging offers both advantages and disadvantages. Firstly, it provides high resolution; the spatial resolution of two-photon imaging is at the micrometer or even submicron level, significantly higher than the hundreds of micrometers level of electrode and fiber recording. This allows for simultaneous structural and functional research at the subcellular level *in vivo*. Secondly, there is signal specificity; unlike the local field potentials recorded by electrodes or the cluster calcium signals recorded by optical fibers, the calcium signals detected by two-photon imaging or synchronous electrophysiological signals originate from individual neurons or even dendritic spines. Thirdly, two-photon imaging allows for precise positioning. The accurate spatial observation of target cells facilitates the recording of signals at the subcellular level of specific structures. However, unlike electrodes and fiber optics, which are blindly inserted, two-photon imaging requires precise operation with stereo positioning to avoid the significant bias that can result from inaccurate targeting. Lastly, in contrast to multi-electrode recording, two-photon imaging, when combined with transgenic animals (Chen Q. et al., 2012) and viral transfection (Chen T. W. et al., 2013), enables the study of neural responses among specific subtypes of neuronal populations. Leveraging these advantages, two-photon imaging has become an invaluable tool for examining the neural responses of behaving mice with high spatial resolution (Table 1).

**Table 1:** Comparison of different recording methods in vivo.

Technique	Recording frequency	Spatial resolution	Long-term recording	Calcium indicator	Applications	Reference
Two-Photon Imaging	40 Hz	Subcellular (Visible)	36 days	GCaMP6	High-resolution imaging at cellular and subcellular levels. Structural and functional research in vivo. Study of specific neuronal subtypes using transgenic animals and viral transfection.	Chen Q. et al. (2012), Chen T. W. et al. (2013)
Multi-Channel Single-Unit Recording	30 kHz	Single-cell (Invisible)	3 months	None	High temporal resolution recording of neural activity. Suitable for studying local field potentials.	Wang et al. (2024)
Fiber Optic Gradient Recording	2,000 Hz	Population cells (Invisible)	~7 days	OGB-1 AM/ GCaMP6s	Recording of population calcium signals. Suitable for large-scale neural activity monitoring.	Yao et al. (2018)

To achieve two-photon imaging at both cellular and subcellular scales in behavioral mice, head fixation and body limitations are essential for ensuring the stability of calcium imaging and electrophysiological recording (Evarts, 1968). Stable head fixation is crucial for accurately monitoring behavioral changes in mice. The behavioral paradigms currently used for two-photon imaging primarily involve head-fixed mice and encompass two types of tasks: simple execution or omission of tasks (O'Connor et al., 2009), and two alternative forced choice tasks (Mayrhofer et al., 2013; O'Connor et al., 2013). The first paradigm involves animals responding to sensory stimuli within a designated time frame to receive rewards, such as water licking or food consumption. Failure to execute the commands within the specified window results in signal warnings. The second paradigm requires animals to make a correct choice between two options based on distinct sensory information, like licking a water outlet on either the left or right side of their chin. The selection of different behavioral paradigms is tailored to various experimental needs, ultimately connecting sensory stimuli with reward behaviors. Under these paradigms, even naïve mice can quickly become adept at the tasks, mastering the associative behaviors within a few days. High-throughput training and imaging experiments conducted over a short period allow scientists to study perceptual behaviors from multiple dimensions under a variety of task conditions (Chen J. L. et al., 2013).

Memory is formed through long-term changes in synaptic efficacy, a process known as synaptic plasticity. Currently, the primary mechanism by which memory is encoded in these neuronal populations is believed to be synaptic strength plasticity, which primarily occurs within the dendritic regions of excitatory neurons. Synapses are considered the smallest unit for memory storage and are increasingly being studied in learning tasks (Kastellakis et al., 2023). Through two-photon imaging techniques, we have gained a deeper understanding of the structural and functional aspects of synapses, particularly regarding the integration of dendrites and molecular mechanisms in the intact brain during behavioral plasticity in animals. In terms of structural plasticity, chronic two-photon imaging has been used to observe the remodeling of dendritic spines, including sensory deprivation (Hofer et al., 2009), changes in dendritic spines at different developmental stages (Lendvai et al., 2000), and behavioral learning (Xu et al., 2009). Studies have shown that short-term monocular deprivation leads to extensive pruning of inhibitory synapses (Chen J. L. et al., 2012). Research indicates that during the initial learning phase, one-third of new dendritic spines appear in clusters, and most of these clusters consist of adjacent spine pairs (Fu et al., 2012). This suggests that repeated activation during learning induces the emergence of new synaptic clustering phenomena.

In terms of functional plasticity, Choi et al. demonstrated (Choi et al., 2018) that the number and size of dendritic spines on CA1 engram cells receiving input from CA3 engram cells labeled under fear conditions were greater than those on non-engram cells in the CA1 region. Hwang et al. demonstrated that after motor learning, dendritic spine density increased and newly formed dendritic spines persisted in primary motor cortex engram cells, while no similar changes were observed in adjacent unlabeled neurons (Hwang et al., 2022). Wright et al. used simultaneous imaging with iGluSnFR3 and RCaMP2 to uncover the mechanisms underlying synaptic function and plasticity in L2/3 motor cortex neurons during *in vivo* learning (Wright et al., 2025). Apical dendrites form task-related functional clusters through high synaptic activity, potentially optimizing signal integration efficiency during motor learning. Neurons exhibit multiple excitability-dependent plasticity rules: apical dendritic plasticity is primarily regulated by local synaptic synergistic activity, while basal dendritic plasticity is heavily dependent on somatic activity.

## 3 Indicators for detecting neural activity

### 3.1 Calcium indicator

Calcium ions (Ca^2+^) participate in a variety of critical functions, including muscle contraction, hormone secretion, and intracellular metabolism, with these processes occurring over a broad timescale ranging from microseconds to several hours within the human body (Berridge et al., 2003). Moreover, Ca^2+^ plays a unique role in the nervous system, including the facilitation of neurotransmitter release through the induction of synaptic vesicle exocytosis. Consequently, Ca^2+^ is a significant contributor to synaptic plasticity. Traditionally, high Ca^2+^ influx was proposed to drive LTP, while moderate Ca^2+^ elevation induced LTD under certain conditions. Synaptic plasticity involves numerous Ca^2+^ regulatory elements, including voltage-gated Ca^2+^ channels (VGCCs), Ca^2+^-permeable N-methyl-D-aspartate receptors (NMDARs), *α*-amino-3-hydroxy-5-methyl-4-isoxazolepropionic acid receptors (AMPARs), and the Ca^2+^ sensor calmodulin (CaM). These components trigger conformational changes to mediate long-lasting plasticity (Mateos-Aparicio and Rodriguez-Moreno, 2020).

In the past two decades, significant efforts have been directed towards capturing ‘real-time’ cellular activity by recording electrochemical events, leading to the development of several technologies based on the principles of action potential. As a result, the primary ions involved in membrane potential—sodium (Na^+^), potassium (K^+^), and calcium (Ca^2+^)—have emerged as potent candidates for the creation of fluorescent indicators. These indicators, when expressed under specific promoters, enable the real-time monitoring of single-cell activity across neuronal populations.

**Figure 1:**
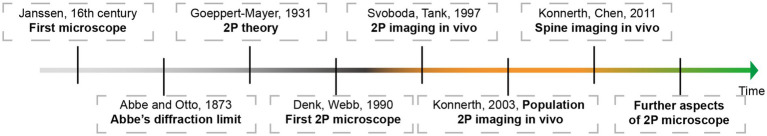
Highlighting milestones of 2P (two photon) microscope development and its applications.

Real-time observation of neuronal excitability can be achieved using calcium indicators, which rely on changes in intracellular Ca^2+^ concentration as an indicator of neuronal activity. The time course and amplitude of Ca^2+^ dynamics are critical biological parameters, given that the calcium concentration in most neurons, typically ranging from 50 to 100 nM, increases tenfold during action potential firing (Berridge et al., 2000). The rapid evolution of genetically encoded calcium indicators (GECIs) in recent years has marked a significant advancement in neuroscience (Tables 2, 3). These tools, including virus-mediated transfection (Kato et al., 2015), intrauterine electroporation of plasmids, and transgenic mice model (Issa et al., 2014), have greatly facilitated the study of large-scale neural networks in behaving animals, as well as the long-term observation of neuronal function and morphology. In the realm of GECIs, a notable development was made by Chen from the Svoboda research group, who developed GCaMP6 in 2013 (Chen T. W. et al., 2013), a calcium indicator that demonstrated high sensitivity (a calcium indicator denotes the magnitude of detectable alteration in its fluorescent signal in response to fluctuations in intracellular Ca^2+^ concentration) and stability at both the single-cell and subcellular levels. The dynamic ranges of purified protein of GCaMP6 sensors are around 38- to 63-fold, superior to those of the previous GCaMP3 (12.3-fold) and GCaMP5 (17.4–32.7-fold) versions. Indeed, the outstanding performance of these sensors has led to their widespread use in virtually all model organisms, and they are highly compatible with a wide range of imaging techniques, including fiber photometry recording, multiphoton imaging, and the recently developed mesoscopic wide-field imaging. Compared to GCaMP5G, GCaMP6s showed 3-fold higher calcium affinity and 1.3-fold higher saturated fluorescence with comparable baseline levels. The GCaMP6f had 2-fold faster rise time and 1.7-fold faster decay kinetics. Their superior performance has enabled broad applications in various imaging techniques (see Figure 1).

**Table 2:** Milestones in the field of Ca^2+^ imaging.

Indicator	Applications	Highlights	Reference
OGB-1 AM	Study of neural networks in living animals.	Introduction of Multicell Bolus Loading technique.	Stosiek et al. (2003)
GCaMP6	Study of specific neuronal subtypes using transgenic animals and viral transfection.	Development of genetically encoded calcium indicators (GECIs). Precise positioning of target cells.	Chen Q. et al. (2012)
GCaMP6	Real-time monitoring of single-cell activity across neuronal populations.	Development of GCaMP6, a highly sensitive and stable calcium indicator.	Chen T. W. et al. (2013)
Cal-520	High sensitivity and low signal-to-noise ratio calcium imaging.	Development of Cal-520, a chemically synthesized calcium indicator.	Tada et al. (2014)
GCaMP7	Functional analysis at subcellular level. Sparse expression and high-brightness tracing of dendritic segments.	Development of GCaMP7b with higher background fluorescence.	Dana et al. (2019)
GCaMP8	Precise tracking of large neuronal populations on relevant timescales.	Development of GCaMP8 with ultra-fast kinetics and high sensitivity.	Zhang et al. (2023)

**Table 3:** Overview of genetically encoded indicators.

GECI	Kd in vitro (nM)	ΔF/F per AP in tissued	Half-decay time (ms)	Reference
GCaMP6f	380	+0.22	140	Chen T. W. et al. (2013)
GCaMP7f	174	+0.23	297	Dana et al. (2019)
GCaMP8f	334	+0.37	87.5	Zhang et al. (2023)
NEMOf	528	+0.80	409	Li R. et al. (2023), Li J. et al. (2023)
jRGECO1a	150	+0.19	200	Dana et al. (2016)

GEVI	ΔF/F (%) (in vitro)	On/off kinetics	In vivo application	Reference
QuasAr3-s	−23 per AP	0.90/0.93 ms	Mice	Adam et al. (2019)
SomArchon	−53 per AP	0.61/1.10 ms	Mice	Piatkevich et al. (2019)
Voltron525	−6.5 per AP	0.64/0.78 ms	Mice, zebrafish, and flies.	Abdelfattah et al. (2019)
ASAP3	−24 per AP	0.98/4.29 ms	Mice	Villette et al. (2019)
JEDI-2P	−18.2 per AP	0.54/1.21 ms	Flies and mice	Liu et al. (2022)
ASAP4b	+35 per AP	1.53/ 1.54 ms	Mice	Evans et al. (2023)
ASAP5	−43 per AP	0.78/1.12 ms	Flies, fish, and mice, human stem-cell-derived neurons	Hao et al. (2024)

GENI	Responses (maximum ΔF/F0)	Affinity (EC50)	In vivo application	Reference
iGluSnFR3	0.54	196 μM	Mouse	Aggarwal et al. (2023)
dLight	2.3	330 nM	Mouse	Patriarchi et al. (2018)
iGABASnFR	3.3	30 μM	Zebrafish, mouse	Marvin et al. (2019)
GRAB_ACh2.0_	2.8	2 μM	Fly, mouse	Jing et al. (2020)
GRAB_DA2h_	2.8	7 nM	Fly, mouse	Sun et al. (2018)
GRAB_NE1m_	2.3	930 nM	Zebrafish, mouse	Feng et al. (2019)
GRAB_5- HT1.0_	2.5	14 nM	Fly, mouse	Wan et al. (2021)
GRAB_ATP1.0_	5.0	~6.7 μM	Zebrafish, mouse	Wu et al. (2022)

At the same time, Thy1-GCaMP6 transgenic mice were developed for neuronal population imaging, which were useful for long-term, high-sensitivity imaging in behaving mice (Dana et al., 2014). For the efficiency of cells expression, as the percentage of responded L2/3 cells for an example, the highly expressing GCaMP6s line (GP4.12, 42.76 ± 11.1%) the fraction was similar to AAV- GCaMP6s (50.96 ± 13.7%). For the GCaMP6f line (GP5.17, 19.56 ± 13.7%) the fraction was also comparable to AAV-GCaMP6f (27.56 ± 17.7%).

Additionally, in the realm of chemically synthesized calcium indicators, Tada et al. made a significant contribution in 2014 by developing Cal-520 (Tada et al., 2014), an indicator known for its high sensitivity and low signal-to-noise ratio. The choice between chemically synthesized and genetically encoded calcium indicators is guided by the specific aims of the experiment.

**Figure 2:**
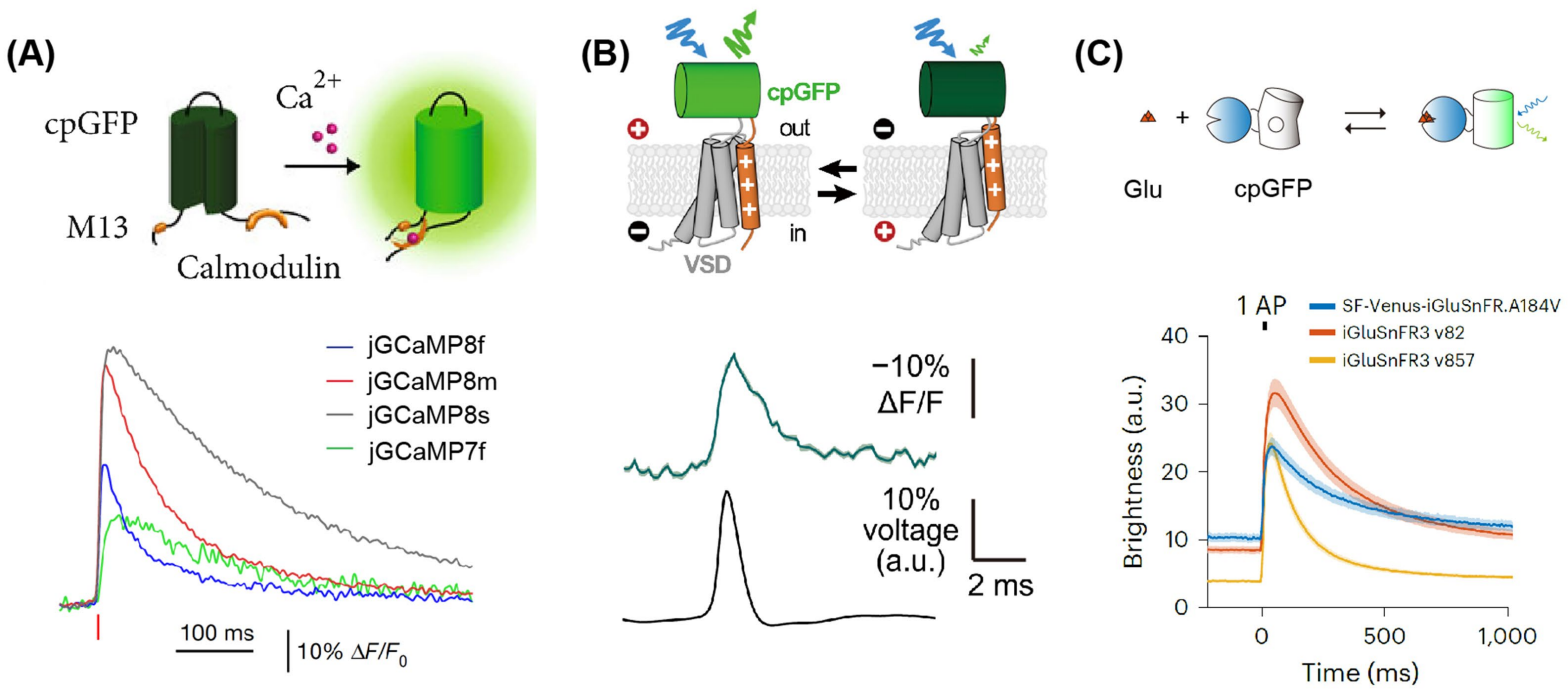
Principles and fluorescence signals of GECI, GEVI, and GENI. **(A)** Upper, the working principle of GECIs (Roth and Ding, 2020). Lower: the signals of GECIs (Zhang et al., 2023). Averaged fluorescence signals are elicited by single APs, with each color representing a distinct sensor. **(B)** Upper, the working principle of GEVIs (Liu et al., 2022). Lower: the signals of JEDI:2P. An example of fluorescence signals elicited by single APs. **(C)** Upper, the principle of glutamate indicators. Lower: the signals of glutamate indicators (Aggarwal et al., 2023). Averaged of fluorescence signals elicited by single APs, each color represented its corresponding sensor. Adapted with permission.

Optical imaging offers a powerful advantage: the capability to simultaneously capture images of multiple colors representing different subtypes of cells. Most image acquisition systems are capable of imaging two colors, typically green and red, and extending this capability to more colors is feasible (Inoue et al., 2019). By integrating two independent recombinase systems, such as cre-loxp and Flp-FRT, it is possible to label distinct neuronal populations, enabling the investigation of their interactions and contributions to behavior. An alternative strategy involves labeling different subtype cells with bio-markers of various colors. A third benefit of optical imaging is the functional analysis at the subcellular level, particularly under two-photon microscopy conditions. This analysis can focus on structures like boutons or dendritic spines, which necessitate sparse expression and high-brightness tracing of dendritic segments. GECIs with higher background fluorescence, such as GCaMP7b (Dana et al., 2019), are well-suited for this purpose. Furthermore, the latest generation of GECIs, like GCaMP8 (Figure 2A), boasts ultra-fast kinetics with half-rise times of 2 ms and unparalleled sensitivity (Zhang et al., 2023). These properties allow for the more precise tracking of large neuronal populations on timescales that are relevant to neural computation. Li et al. replaced the widely used cpGFP with the brighter fluorescent protein mNeoGreen, which they have dubbed the “NEMO” GECI (Li J. et al., 2023). Compared to the latest GCaMP8s or the most widely used GCaMP6s, NEMO exhibits higher sensitivity and signal-to-noise ratio for intracellular calcium signals, with a response amplitude approximately 10 times higher, and a response speed comparable to GCaMP6f.

However, calcium signal recording does not accurately reflect the input information carried by dendrites with high fidelity. This limitation is since the response time of a neuron’s action potential is about one millisecond (Bradley et al., 2009; Scanziani and Hausser, 2009), while the subsequent calcium response can last for tens of milliseconds (Podor et al., 2015). At high frequencies of action potential firing, calcium responses can overlap, complicating data analysis. Accurately monitoring the firing patterns and frequencies of action potential is challenging, and detecting the subthreshold electrical activity of neurons is even more so. These difficulties significantly limit the study of information integration mechanisms (Quan et al., 2010).

### 3.2 Voltage-sensitive indicator

Since the mid-1970s discovery of optical dyes that represent changes in membrane potential (Cohen et al., 1974), voltage-sensitive indicators have become a powerful tool for imaging neural activity. These indicators offer a more direct response to neuronal activity compared to the second messenger, calcium ions, and provide a shorter time-window advantage for detecting action potentials. Typically, neurons have a resting potential of −60 mV relative to the extracellular potential. Upon action potential firing, the intracellular potential rises from −60 mV to +40 mV, allowing for the detection of subthreshold and hyperpolarization potentials. Notably, this potential change is largely confined to the 5 nm-thick cell membrane, where a ~ 100 mV change during AP firing corresponds to an electric field change of 2 × 10^5^ V/cm (Brinks et al., 2015). Consequently, a significant challenge for voltage-sensitive indicators is their limited area of photoreception. To address this, there is a need for faster scanning speed imaging systems and brighter fluorescent probes to counteract photobleaching.

Genetically Encoded Voltage Indicators (GEVIs) are designed based on the property of voltage-sensitive indicators and typically consist of a voltage-sensing domain coupled with a fluorescent group (Lin and Schnitzer, 2016). In recent years, several research groups have developed the next generation of voltage indicators, including QuasAr3-s (Adam et al., 2019), SomArchon (Piatkevich et al., 2019), ASAP3 (Villette et al., 2019), ASAP4 (Evans et al., 2023), JEDI-2P (Liu et al., 2022) and Voltron525 (Abdelfattah et al., 2019). QuasAr3-s, and SomArchon are the latest GEVIs based on opsin-based proteins, offering a high temporal resolution for representing individual action potentials. However, these GEVIs have a major drawback: their insufficient brightness, which necessitates an increase in laser excitation power and can result in photobleaching. Additionally, due to their absorption characteristics, these indicators are not suitable for two-photon imaging.

In GEVI, the ASAP family related to the voltage-sensitive domain (VSD) is based on the fusion of cpGFP to achieve voltage imaging. The latest sensors in this family, namely ASAP3 or ASAP4, are exceptionally well-suited for *in vivo* two-photon imaging. Villette and colleagues successfully applied somatically localized ASAP3-Kv and ULoVE two-photon microscopy to awake, head-fixed mice, detecting action potential (AP) and subthreshold voltage dynamics in cortical and hippocampal neurons over periods ranging from minutes to days (Villette et al., 2019). Although their dynamics are slightly slower compared to opsin-based GEVIs, these sensors can discern individual action potentials and subthreshold potentials *in vivo*, with fluorescence changes of approximately 5–10%. They are regarded as some of the most potent tools for *in vivo* two-photon research on neuronal voltage signals. Furthermore, with JEDI-2P, the authors have reported an unprecedented capability to capture voltage dynamics in deep cortical layers over durations that exceed 40 min (Figure 2B). This development paves new ways to unravel the mysteries of neural circuits during complex behavioral tasks. Michael Z. Lin’s research group developed a new generation ASAP, ASAP5, which exhibits significantly enhanced activation kinetics and response sensitivity near resting membrane potential, enabling more accurate detection of action potential (AP) firing and subthreshold activity. When detecting APs in vivo, ASAP5 exhibits a higher signal-to-noise ratio (SNR) than previous GEVIs. Additionally, it can detect mEPSPs in cultured human-induced neurons, successfully capturing synaptic quantum events (Hao et al., 2024).

### 3.3 Neurotransmitter indicators

Monitoring the action potential activity of neurons is arguably the most direct method for observing information transmission in the brain. However, the communication between neurons is primarily chemically mediated, occurring through neurotransmitters such as glutamate and GABA, as well as neuromodulators like dopamine, acetylcholine, norepinephrine, and serotonin (5-HT). These substances are integral to the physiological and pathological processes underlying information transmission.

Recently, there has been significant progress in the development of fluorescent indicators for monitoring neurotransmitters, neuromodulators, and second messengers in downstream intracellular signaling pathways (Table 3). One strategy, based on periplasmic binding proteins (PBPs), involves the insertion of fluorescent proteins into glutamate-binding proteins. The presence of PBPs on the outer surface of the cell membrane allows any changes in FRET (Förster Resonance Energy Transfer) efficiency to reflect changes in extracellular glutamate concentration. Another approach, inspired by the GECI based on cpGFP (a circularly permuted green fluorescent protein), fuses cpGFP with proteins that bind to specific neurotransmitters, namely Genetically Encoded Neurotransmitter/ Neuromodulator Indicators (GENIs). The binding of these neurotransmitters induces conformational changes that, in turn, affect the fluorescence of cpGFP. The latest generation, iGluSnFR3 (Aggarwal et al., 2023), exhibits faster dynamics—approximately twice as fast as its predecessor, SF-iGluSnFR (Marvin et al., 2018)—and demonstrates higher sensitivity under two-photon excitation, being 20 times more sensitive than the previous generation (Figure 2C).

The development of the above probes has paved the way for a series of indicators for detecting neurotransmitters and neuromodulators, such as dopamine indicators (“GRAB_DA_” (Sun et al., 2018) and “dLight” (Patriarchi et al., 2018)), iGABASnFR (Marvin et al., 2019), acetylcholine (Jing et al., 2020), norepinephrine (Feng et al., 2019), and 5-HT (Wan et al., 2021). These indicators typically bind to specific G protein-coupled receptors (GPCRs), such as the D1 or D2 dopamine receptors of dopamine indicators, with cpGFP inserted into the intracellular loop of GPCRs. To measure downstream intracellular signaling pathways activated by neuromodulators, several groups have recently developed fluorescent kinase indicators, such as those that can detect changes in protein kinase A activity (Zhang J. F. et al., 2021; Ma et al., 2018) or in the activity of the cyclic AMP responsive element binding protein (Laviv et al., 2020). Li Yulong’s research group has developed HaloDA1.0, a far-red light dopamine indicator (Zheng et al., 2025). By combining it with green and red fluorescent indicators, HaloDA1.0 enables researchers to simultaneously capture changes in multiple neurochemical transmitters, providing insights into their spatiotemporal dynamics and regulatory relationships. This breakthrough overcomes the challenge of simultaneously detecting multiple neurochemical substances *in vivo*, providing powerful support for elucidating the complex neurotransmitter regulatory networks in the brain.

## 4 Multiple two-photon imaging methods: from head-fixed to freely moving imaging paradigms

### 4.1 Classic two-photon imaging in head-fixed behaving mice

**Figure 3:**
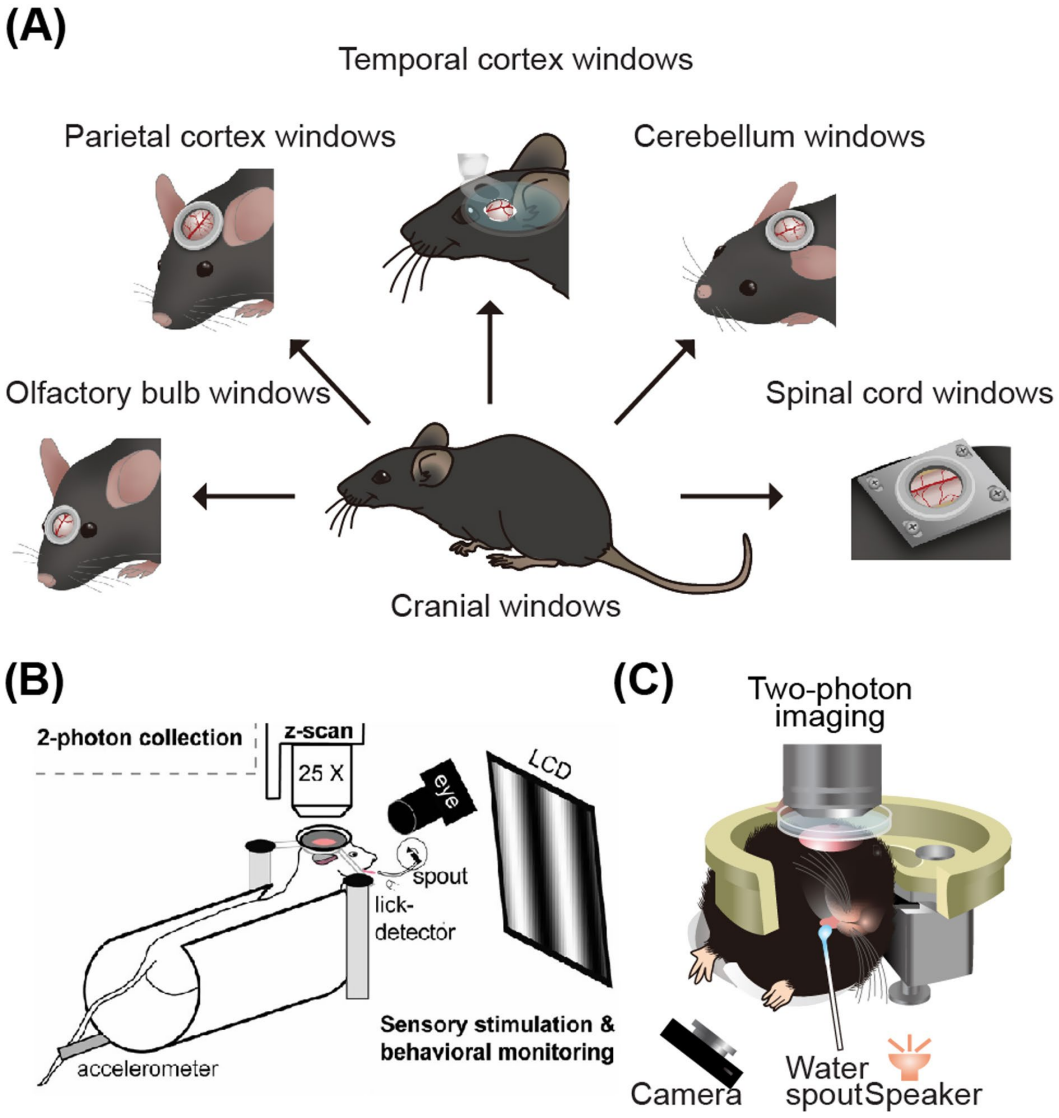
Schematic diagram of two-photon imaging of the parietal and lateral brain areas. **(A)** CNS windows for imaging. From left to right: olfactory-bulb window, parietal-cortex window, temporal-cortex window, cerebellar window, and spinal-cord window (Yeon et al., 2022). **(B)** Two-photon calcium imaging on visual cortex of head-fixing mice (Andermann et al., 2010). **(C)** Illustration of two-photon imaging on the auditory cortex of mice while they engage in auditory-related water reward task (Li et al., 2018). Adapted with permission.

By labeling a group neurons with calcium indicators in the target area, it is possible to observe neural network-level activity in behavioral mice with head-fixed apparatus. When observing cortical activity, although the head is fixed, the mouse’s body and limbs can still move. This setup provides the possibility for studying the cortex of somatosensory, motor, visual. Auditory, and other areas (Yeon et al., 2022) (Figure 3A). Depending on the area of study, there are differences in the choice of behavioral paradigms and fixation methods, with distinct approaches for the medial (parietal, Table 4) and lateral brain areas (Table 5).

**Table 4:** Representative publications for recent 2P imaging on the parietal brain area.

Technique	Recording area	Applications	Highlights	Reference
Spherical treadmill with low friction	Somatosensory/motor cortex	Analysis of movement speed, direction, and cortical neural activity during locomotion	Stabilized imaging by reducing brain movement; simultaneous tracking of behavior and neural activity	Dombeck et al. (2007)
3D population imaging	Visual cortex	Imaging during visual-related licking and water-reward tasks	Simultaneous multi-focal-plane imaging; offline 3D reconstruction of structure and function	Andermann et al. (2010)
Olfactory-based behavioral paradigm	Motor cortex	Study of neuron activity during odor discrimination and water-licking tasks	Combined olfactory stimulation	Komiyama et al. (2010)
Whisker stimulation and licking behavioral paradigm	Motor cortex	Investigation of the role of dendritic nonlinear integration in information transformation in the motor cortex	Expanded understanding of neural activity to the subcellular level in behaving mice	Xu et al. (2012)
Targeted electrophysiological recording	Somatosensory cortex	Conducted on target neurons to reveal the membrane potential dynamics specific to labeled neurons	Technical difficulty focuses on the extreme stability of electrophysiological recording by reducing brain movements in behavioral animals	Yamashita and Petersen (2016)

**Table 5:** Representative publications for recent 2P imaging on the lateral brain area.

Technique	Applications	Highlights	Reference
Two-Photon Imaging	Investigation of electrophysiological mechanisms of different neuron types.	Use of tilted objective for complex tasks like spherical treadmill movement.	Issa et al. (2014), Kato et al. (2017), Kuchibhotla et al. (2017), Drieu et al. (2025)
Two-Photon Imaging	Long-term memory storage in holistic bursting cells.	Rotating mouse head method for traditional upright two-photon imaging systems. Ideal for horizontal recording chamber with liquid medium.	Li et al. (2018), Li R. et al. (2023), Huang et al. (2023)

#### 4.1.1 Two-photon imaging of the parietal brain area in behavioral mice

Dombeck et al. (2007) developed an ingenious method for stabilizing imaging during the study of neural activity in the parietal brain areas of mice, such as the somatosensory and motor cortices during movement. They designed a polystyrene foam ball to be placed under the mouse’s body, allowing it to rotate with the mouse’s movements. A camera was used to accurately track the floating ball’s movement. The spherical treadmill, which had low friction, mostly supported the weight and shaking of the running mouse, providing more stable imaging. This setup enabled the analysis of movement speed, direction, and neural activity in the cerebral cortex, facilitating the study of the correlation between these factors. Andermann et al. (2010) introduced the method of 3D population imaging in the imaging experiment during visual-related licking and water-reward tasks (Figure 3B). This 3D imaging technique involves capturing simultaneous images at multiple focal planes, tens of micrometers depth, and reconstructing the 3D structure and function through offline analysis. Moreover, Komiyama et al. designed a behavioral paradigm based on olfactory stimulation and water-licking discrimination tasks (Komiyama et al., 2010) to study the local neural microcircuits in motor cortex neuron activities during behaving tasks. Xu et al. (2012) used a behavioral paradigm related to whisker stimulation and licking tasks to investigate the role of dendritic nonlinear integration in information transformation in the motor cortex. This research expanded the understanding of neural activity to the subcellular level in behaving mice (Figure 3B). Piet et al. (2024) revealed how different strategies employed by animals in visual decision-making tasks influence the activation mechanisms of brain circuits in visual cortex. The results indicate that enhanced activation of the VIP-SST inhibitory circuit in visual comparison strategies contributes to task-adaptive responses.

The above brain areas are all located in the parietal brain area, which is easier to prepare and has a simpler imaging method compared to the lateral and deep brain areas. Studying the electrical activity of individual neurons in behavioral mice is extremely important for a deeper understanding of brain functional mechanisms (Petersen, 2017). Combined with two-photon microscopy targeted electrophysiological recording can be specifically studied on target neurons to reveal the membrane potential dynamics specific to labeled neurons. The technical difficulty focuses on the extreme stability of electrophysiological recording by reducing brain movements in behavioral animals. Yamashita and Petersen achieved electrophysiological recording of targeted neurons in the somatosensory cortex of behaving mice in Yamashita and Petersen (2016). This makes it possible to conduct targeted electrophysiological research on specific types of neurons, enabling the exploration of deeper electrophysiological mechanisms of neurons in behavioral states.

#### 4.1.2 Two-photon imaging of lateral brain areas in behavioral mice

The study of the lateral brain area, particularly in the auditory cortex of awake, behaving mice, presents unique challenges. It often necessitates the removal of a significant number of muscles near the ear and the separation of temporal blood vessels during surgery. Additionally, the process of tilting the objective or rotating the mouse’s head during imaging can introduce a lag, making the study of the lateral brain area more complex than that of the top brain area. Recently, there have been few reports on two-photon imaging in the auditory cortex of awake, behaving animals (Issa et al., 2014; Deneux et al., 2016; Li et al., 2017). Issa et al. plotted the distribution of sound frequency response on multi-scale auditory cortex imaging of awake animals in Issa et al. (2014). Kato et al. (2017) and Kuchibhotla et al. (2017) performed two-photon functional imaging and blind electrophysiological recording in awake (or behaving) mice, respectively. In addition, by two-photon long-term imaging and optogenetic techniques, Drieu et al. (2025) discovered that the auditory cortex (AC) not only participates in sensory processing but also performs advanced computations, driving rapid learning and performance improvement. Although reinforcement feedback is essential, it masks the animal’s rapid mastery of task rules. The tilted objective can enable mice to perform complex tasks such as the movement of a spherical treadmill in the upright state, see Figure 3C (Li et al., 2018). These studies have significantly promoted our understanding of the electrophysiological mechanisms of different types of neurons in the lateral brain area.

Alternatively, Li et al. found a candidate of engram, namely holistic bursting cells (exhibit high-frequency burst firing and holistic coding responses to complex sounds), stored long-term memory in the auditory cortex by the method of rotating the mouse head (Li R. et al., 2023). While this method can increase discomfort and struggle in mice, adaptive training can be added to alleviate these issues. The advantages of the rotating mouse head method are below: firstly, it is widely used and suitable for traditional upright two-photon imaging systems; secondly, it is beneficial for conducting further applications in locally targeted population neurons (Huang et al., 2023). This is because the imaging cortex is parallel to the horizontal plane, making it ideal for placing a horizontal recording chamber filled with a liquid medium, which is beneficial for electrophysiological and electroporated experiments.

### 4.2 Miniature two-photon imaging in freely behaving mice

Traditional two-photon imaging systems, with their complex optical paths, pose challenges for recording in freely moving animals. The main difficulties in adapting these systems for such use include the heavy weight of the imaging system and the significant motion artifacts that occur during free movement. To address these challenges, scientists have conducted extensive research in the field of miniaturized two-photon technology. In 2001, the Denk research group achieved relatively stable two-photon imaging in the somatosensory cortex of rats, taking the first step in the development of a miniature two-photon imaging system (Helmchen et al., 2001). However, the system had a slow scanning speed (0.5–2 Hz) and was too cumbersome, weighing 25 g, which is comparable to the weight of an 8-week-old mouse (~25 g). Flusberg et al. (2005) reported a mouse deep brain imaging device weighing approximately 3.9 g and successfully achieved imaging, but the motion artifacts of the animals were very obvious during free movement. In addition, the Schnitzler (Piyawattanametha et al., 2009) and Kerr research groups (Sawinski et al., 2009) made improvements to the system’s resolution and scanning speed of the micro two-photon microscope, respectively.

Zong et al. (2017) developed a FHIRM-TPM (fast, high-resolution, miniaturized two-photon microscope) that was lighter in weight (2.15 g) and offered superior resolution and scanning speed. Furthermore, in 2021, the research team developed the second generation FHIRM-TPM (Zong et al., 2021), which significantly increased the imaging field of view (420 × 420 μm^2^) compared to the previous generation. This system enabled 1-mm-depth volume imaging, achieving high-quality structural and functional imaging of cell populations and subcellular structures in the visual cortex. In 2023, the third-generation miniaturized three-photon microscope was developed, achieving functional imaging of neurons in the entire cerebral cortex and hippocampus of free-moving mice (Zhao et al., 2023). Liu et al. (2025) develop a platform for 2P fiberscopes, enabling near-zero rotational burden during 2P neural imaging in freely behaving mice. The development of this epoch-making miniature two-photon microscope is expected to greatly facilitate the exploration of neural dynamics within the neural circuits of freely moving mice. Moreover, if the miniature two-photon microscope (2 PM) system can be open-sourced, it will help broaden the application of such imaging instruments and greatly assist in single-cell and subcellular-level research on freely awake animals.

### 4.3 Real-time imaging of multiple brain areas

**Figure 4:**
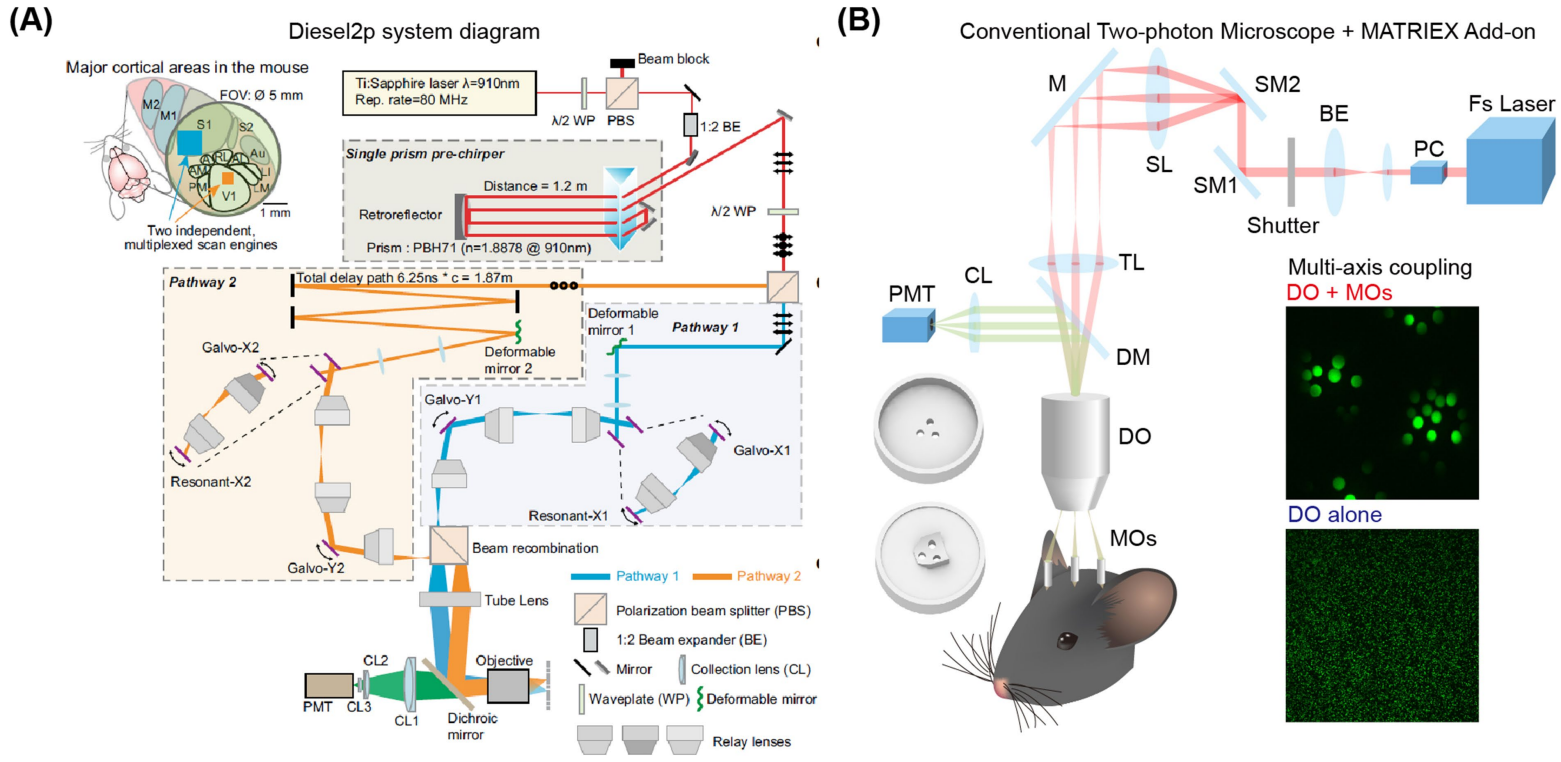
Schematic diagram of two-photon imaging of multiple brain areas. **(A)** Characteristics and layout of the Diesel2p system. Left: On the left, a field of view (FOV) of Ø5mm can cover multiple brain regions, while an independent scanning engine can simultaneously capture ongoing neural activity in multiple cortical regions through optimized scanning parameters. Right: Schematic diagram of the Diesel2p system setup (Yu et al., 2021). **(B)** Experimental diagram of the MATRIEX imaging system (Yang et al., 2019). The two round 3D objects in the lower-left corner are the top and bottom views of the mouse head chamber used for *in vivo* imaging. DO: dry objective; MOs: miniaturized objectives. The insert photo indicates an illustration of the two-stage magnification and multi-axis coupling. Adapted with permission.

Traditional two-photon microscopes are primarily designed for real-time imaging within a specific range (less than 1 mm), which limits the studies to a single brain area. However, since most behaviors involve interactions across multiple brain areas, research focusing on a single area is becoming increasingly limited. To overcome this limitation, several research groups have made significant breakthroughs in the field of multi-area brain imaging. In terms of expanding the axial field of view, the Bar-Noam et al. (2016) and Grewe et al. (2011) research groups have extended the limit by scanning multiple focal planes at different focal depths. Meanwhile, the Ji Na research group has utilized a Bessel-shaped laser beam with an extended focus function to image all planes within a specific depth range (Lu et al., 2017). Regarding the expansion of the horizontal field of view, three research groups have made notable advancements. Luo Liqun’s research group achieved simultaneous imaging of both large and small brains using two modified two-photon systems (Wagner et al., 2019). The Smith research group first divided the laser beam and extended the optical path of one beam to achieve different excitation time domains in different brain areas (Figure 4A). Subsequently, they used an additional polarizer to simultaneously image any two brain areas within the scanning field of view (Stirman et al., 2016; Yu et al., 2021). In the two-photon imaging of two brain areas, the Schnitzer research group divided the laser into two beams and combined them with the lens of a small microscope to achieve imaging of the two brain areas (Lecoq et al., 2014).

Among these three methods, the approach by the Luo Liqun group requires two modified two-photon systems. The Smith research group’s method is limited to selecting two brain areas within a 3.5 mm diameter. The Schnitzer research group’s technique necessitates performing scans at a rate of 1 Hz on a scale of 2.5 mm or larger. These methods, while innovative, have certain limitations. They typically rely on highly specialized electronic devices and optical components, which can be expensive. Additionally, it is challenging to observe any area of the cerebral cortex simultaneously. To address these challenges, Yang et al. successfully combined a novel method of ‘two-stage amplification and multi-axis optical coupling’ to achieve simultaneous imaging of multiple brain areas at different depths (Yang et al., 2019). This approach utilizes low-magnification air objectives along with multiple micro-objectives immersed in water. It builds upon traditional single-axis two-photon microscopy to enable simultaneous imaging of multiple brain areas with completely different coordinates distributed in both the axial and lateral directions—separated by more than 1 mm and extending up to 12 mm (Figure 4B). This advancement is expected to greatly facilitate the study of three-dimensional whole-brain neural circuit dynamics at the single-cell resolution level.

### 4.4 Deep brain imaging

**Figure 5:**
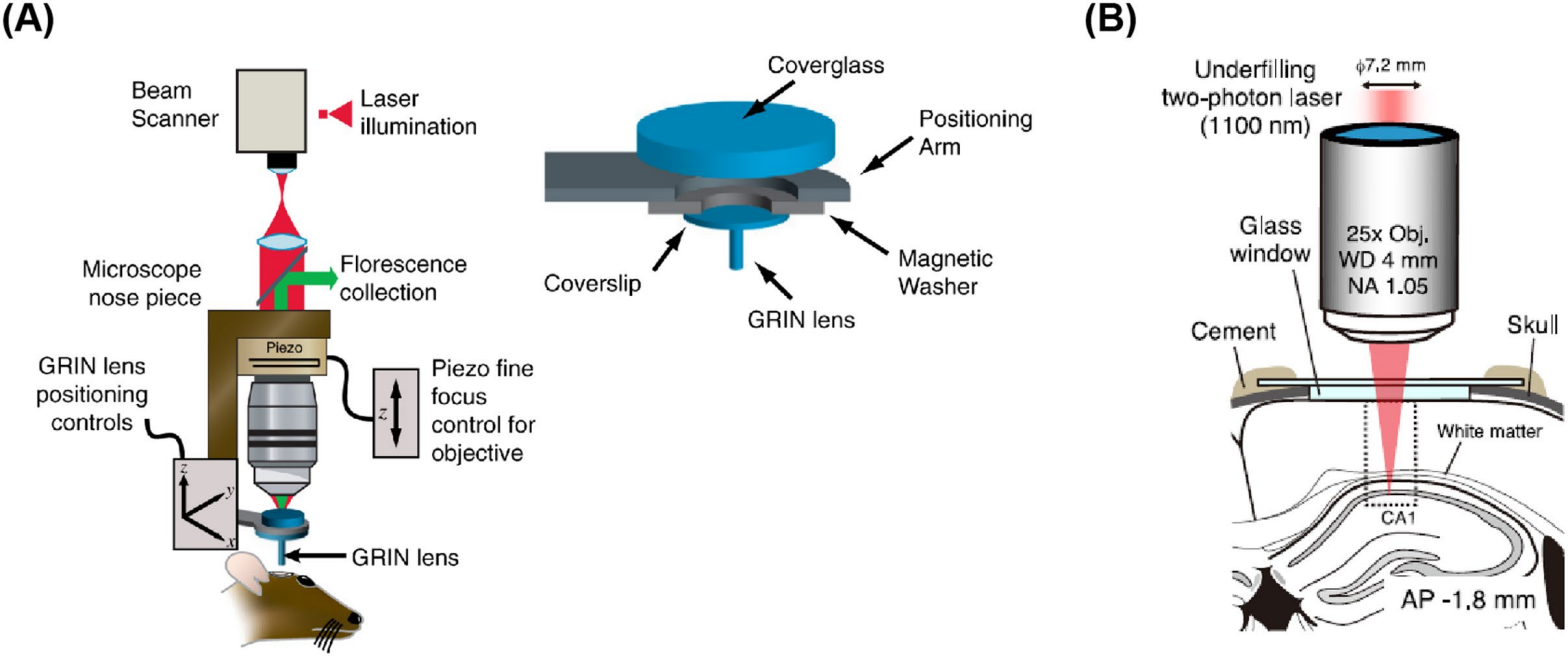
Schematic diagram of two-photon imaging of deep brain areas. **(A)** GRIN lens under coverslip implantation apparatus (Murray and Levene, 2012). **(B)** Shows in vivo calcium imaging of intact hippocampal CA1 region (Kondo et al., 2017).

Currently, the maximum detection depth for most two-photon imaging systems is less than 1 mm, which restricts research on neural activity in deeper brain regions. To date, three main approaches have been developed for studying these deep brain areas: firstly, improving surgical techniques; secondly, by using appropriate gradient index (GRIN) lenses to relay the imaging optical path of traditional two-photon microscopes; and thirdly, improving imaging systems and fluorescent indicators. In terms of surgical innovation, Dombeck et al. (2010) successfully recorded calcium signals from individual neurons in the hippocampus by removing the overlying cortex. This procedure allows the imaging system to directly observe the hippocampus, facilitating the study of neural activity related to decision-making and navigation behaviors. Murray and Levene (2012) pioneered the integration of GRIN lenses into two-photon imaging systems. They implanted GRIN lenses into deep brain areas, enabling the two-photon imaging system to observe neural activity in these areas (Figure 5A). The Matsuzaki research group extended the detector depth of two-photon systems to deep areas such as the prefrontal cortex and hippocampus in Kondo et al. (2017). Their innovation involved using an objective lens with an NA value of 1.0 and a laser with a wavelength of 1,100 nm to excite red fluorescent indicators, allowing the study of these regions (Figure 5B). This non-invasive deep brain imaging with two-photon microscopy is poised to revolutionize the field by illuminating various areas of the brain. Hainmueller et al. (2024) indicate unique roles for parvalbumin- and somatostatin-positive interneurons in the dentate gyrus that are distinct from those in CA1-3 and may support routing of novel information. In conclusion, as optical methods for deep brain imaging continue to evolve, neuroscientists will be able to delve deeper into the study of these regions, unraveling the mysteries of neural circuits.

## 5 Summary and outlook

In summary, breakthroughs in research on various brain areas in mice engaged in behavior are arriving at a rapid pace. However, the paradigms currently used tend to be relatively simple and reductionist. In fact, the behavior is complex and variable and evolves. Therefore, it is essential to observe the neural responses of a subgroup of neurons under behavioral tasks in multiple dimensions—both temporally and spatially—and to analyze perceptual behavior from a more holistic perspective. In 2015, the Svoboda research group reported a large-scale activity map of somatosensory cortex neurons engaged in the whisker-based object localization behavior paradigm (Peron et al., 2015). By recording the calcium activity of over 12,000 neuronal populations, they revealed the correlation between the activity of corresponding somatosensory cortex neuron populations during the mice’s tactile behavior. This discovery provides a robust method for simultaneously observing the responses of a larger number of neurons during corresponding behaviors at a large scale.

Similarly, optical imaging methods with higher temporal resolution enable more in-depth research on the subcellular level of neurons in behaving mice, allowing for further investigation into the electrophysiological signals of targeted neurons. In the realm of the hardware and dye innovations of two-photon microscopy, the three-photon microscope has also seen rapid development, particularly for deeper-scale observations. In 2018, Xu’s research group made significant advancements by developing a three-photon microscope imaging method. This method allows for the observation of layer 4 neurons in adult mice under an intact skull, thereby avoiding the physical and chemical changes that surgery on local brain tissue might cause. It brings the study of real neural tissue closer to its natural state, revolutionizing the non-invasive observation of brain neurons (Wang T. et al., 2018). Additionally, Kerr’s research team has developed a new type of head-mounted three-photon microscope, which can be used for imaging deep cortex in freely moving rats. It enables researchers to observe the neural signals in depth >1.1 mm of free-moving rats performing tasks, offering a powerful new tool for studying the deep cortex (Klioutchnikov et al., 2023).

The capacity to optically record the electrical activity of individual neurons in behaving animals significantly advances our comprehension of the mechanisms of information transmission within the nervous system. Animal behavior is generated by neuronal activity patterns that encompass a wide range of temporal and spatial scales. Therefore, to understand how neural circuits during behavior, it is imperative to long-term record the excitability activities of neuronal populations with a high temporal resolution (~1 ms). While GECIs are typically employed to monitor the activity of a multitude of neurons, they measure action potentials inaccurately due to the slow dynamics of calcium signals. This limitation is particularly evident when sub-threshold voltage signals remain undetected (Svoboda et al., 1997). Utilizing GEVIs for voltage imaging can surmount these hurdles, facilitating the visualization of rapid spikes and subthreshold dynamics within genetically specified neurons (Lin and Schnitzer, 2016). The demands for high imaging speed and excitation intensity required for voltage imaging, combined with the smaller indicator locating cell membrane volume, set more stringent standard imaging setup for voltage indicators compared to GECI. GEVI can reveal non-powered electrical activity and solve the labeling time with sub-millisecond resolution rather than GECI (Lin and Schnitzer, 2016). There is an urgent need for the development of two-photon microscopy with a faster scanning speed and a higher signal-to-noise ratio (Li R. J. et al., 2023).

With technological advancements in calcium imaging, substantial challenges persist in managing and analyzing large-scale datasets. For instance, mesoscopic two-photon microscopy yields up to 4 GB of data per minute (Li R. et al., 2023), demanding robust computational infrastructure. Voltage imaging exacerbates this issue, generating 8 GB/min with higher frame rates, while signal complexity—including overlapping action potentials and neighboring neuronal fluorescence—complicates accurate activity inference (Zhang X. M. et al., 2021). Standardization and sharing of such data remain problematic due to inconsistent formats, hindering collaboration and reproducibility. Data analysis pipelines typically involve motion correction, neuron identification (via manual ROIs or automated algorithms), action potential detection, and cross-session registration. While tools like calcium imaging algorithms have been developed, higher firing rates (over ~25 Hz) challenge spike inference due to sensor kinetics (Inoue et al., 2019). Collaborative efforts to establish universal standards and user-friendly analytical methods are crucial to leveraging big data’s potential across disciplines, addressing both technical bottlenecks and interdisciplinary research needs.

In the future, propelled by the explosive growth of various experimental technologies, an array of new tools will emerge, aimed at decoding perceptual behaviors and progressively reconstructing them across multiple scales, from the entirety of behavior to each pivotal behavioral node. Initially, in terms of morphology, the integration with the f-MOST system (Li et al., 2010), will enable us to trace the comprehensive brain projection map of specific neurons in behavioral states. This will facilitate the study of the upstream and downstream brain regions that may interact with these neurons during the execution of specific behaviors (Wang et al., 2020). Subsequently, genetically encoded viruses will pave the way for optogenetic recording and manipulation of specific subtypes of neurons. Furthermore, targeted two-photon optogenetic manipulation at the micrometer scale can be conducted on individual neurons to observe the behavioral effects of such manipulation (Emiliani et al., 2015). Interventions targeting specific receptors for dendritic function at the subcellular level of neurons can be achieved through a light-controlled drug release system to study functional changes at the subcellular level in behavioral mice. Ultimately, RNA sequencing (Liu et al., 2017) can be applied to active neurons associated with behavioral states, thereby further elucidating changes in gene expression profiles and the genetic underpinnings of such behavior-specificity. With the advancement of these technological tools, two-photon imaging will become faster, deeper, wider and more detailed in exploring the brain. These neuroscience knowledge will bring new ideas to clinical medicine and artificial intelligence.
